# First Insights into the Genome Sequence of Clostridium thermopalmarium DSM 5974, a Butyrate-Producing Bacterium Isolated from Palm Wine

**DOI:** 10.1128/genomeA.00338-18

**Published:** 2018-04-26

**Authors:** Anja Poehlein, Eva Hettwer, Lennart Mohnike, Rolf Daniel

**Affiliations:** a Genomic and Applied Microbiology & Göttingen Genomics Laboratory, Institute of Microbiology and Genetics, Georg-August University of Göttingen, Göttingen, Germany; b Members of the Applied Bioinformatics in Microbiology Course of the Microbiology and Biochemistry M.Sc./Ph.D. program, Georg-August University of Göttingen, Göttingen, Germany

## Abstract

Clostridium thermopalmarium is a moderate thermophilic, rod-shaped, and endospore-forming bacterium, which was isolated from palm wine in Senegal. Butyrate is produced from a broad variety of sugar substrates. Here, we present the draft genome sequence of C. thermopalmarium DSM 5974 (2.822 Mb) containing 2,665 predicted protein-encoding genes.

## GENOME ANNOUNCEMENT

Clostridium thermopalmarium DSM 5974 is an anaerobic, spore-forming, and rod-shaped (0.7 to 1.0 µm wide and 2.0 to 8.0 µm long) bacterium, with optimal growth temperatures between 50 and 55°C and an upper growth limit of 60°C (1). C. thermopalmarium DSM 5974 was isolated from palm wine in Casamance (south Senegal). The strain ferments sugars, resulting in the production of mainly butyrate (approximately 1 mol per mol glucose consumed). In addition, H_2_, CO_2_, acetate, lactate, and ethanol are formed in minor amounts. The bacterium exhibits Gram-positive cell wall features, including a surface layer; however, Gram staining results in a negative type (1).

DNA isolation was conducted using the MasterPure DNA purification kit (Epicentre, Madison, WI, USA). The purified DNA was used to generate Illumina paired-end sequencing libraries. Sequencing was performed using a MiSeq instrument and the MiSeq reagent kit version 3, according to the instructions of the manufacturer (Illumina, San Diego, CA, USA). For quality trimming, Trimmomatic version 0.36 (2) was applied and resulted in 2,538,866 paired-end reads. SPAdes version 3.11.1 (3) was used for genome sequence assembly. A total of 74 contigs (>500 bp) were derived, with an average coverage of 215-fold. Assembly and read coverage validation were conducted with Qualimap version 2.2.1 (4). Afterwards, gene prediction was performed using Prokka (5). The draft genome sequence (2.822 Mbp) harbored 2,665 predicted protein-coding genes, 2 repeat regions, 12 rRNA genes, and 69 tRNA genes. The G+C content of the genome is 30.8%. Putative functions could be assigned for 2,031 of the protein-coding genes. The genome contained putative genes encoding 11 drug resistances and 17 genes associated with prophages.

With respect to the production of butyrate, 7 genes encoding proteins contributing to butyrate metabolism were found in the draft genome, including putative genes coding for acetyl-coenzyme A (acetyl-CoA) acetyltransferase, 3-hydroxybutyryl-CoA dehydrogenase, and an enoyl-CoA dehydrogenase. In comparison to the butyrate producer Clostridium thermobutyricum (6, 7), C. thermopalmarium is able to metabolize sucrose, which is indicated by a sucrose-6-phosphate hydrolase-encoding gene. Furthermore, ethanol can be produced by C. thermopalmarium, indicated by the presence of several genes encoding aldehyde-alcohol dehydrogenases. Acetate and lactate side product formation is driven by genes encoding acetate kinase, phosphate acetyltransferase, and l-lactate dehydrogenase.

### Accession number(s).

The whole-genome shotgun project has been deposited at DDBJ/EMBL/GenBank under the accession number PVXN00000000. The version described here is version PVXN01000000.
